# Testing of tissue specimens obtained from SARS-CoV-2 nasopharyngeal swab-positive donors

**DOI:** 10.1007/s10561-023-10119-8

**Published:** 2023-11-23

**Authors:** Melissa A. Greenwald, Shabnam Namin, Jan Zajdowicz, Alyce Linthurst Jones, Linda Fritts, Matthew J. Kuehnert, Christopher J. Miller, Gregory Ray

**Affiliations:** 1 Donor Alliance, Denver, CO USA; 2 Uniformed Services University of the Health Sciences, Bethesda, MD USA; 3 Vivex, Miami, FL USA; 4 42Bio, Gainesville, FL USA; 5 Allosource, Centennial, CO USA; 6 LifeNet Health, Virginia Beach, VA USA; 7 University of California, Davis, Davis, CA USA; 8 MTF Biologics, Edison, NJ USA; 9 Hackensack Meridian School of Medicine, Hackensack, NJ USA; 10 University of California, Davis, Davis, CA USA; 11 RTI Surgical, Alachua, FL USA

**Keywords:** Tissues, Tissue donor, SARS-CoV-2, Transplantation, Infection, RNA, Viral, Tendons, Fascia lata, Bone, Heart valves, Vascular tissue, Dermis, Musculoskeletal tissue

## Abstract

Risk for transmission of SARS-CoV-2 through allogeneic human tissue transplantation is unknown. To further evaluate the risk of virus transmission, tissues were obtained from deceased donors who had tested positive for SARS-CoV-2 RNA via nasopharyngeal swab. This study evaluated an array of human tissues recovered for transplantation, including bone, tendon, skin, fascia lata, vascular tissues, and heart valves. Tissue samples and plasma or serum samples, if available, were tested for viral RNA (vRNA) using a real time PCR system for the presence of virus RNA. All samples were tested in quadruplicate for both subgenomic (sgRNA) and genomic (gRNA) RNA encoding the SARS-CoV-2 nucleocapsid gene. Amplification of a cellular housekeeping gene served as the positive control for every sample. A total of 47 tissue samples from 17 donors were tested for SARS-CoV-2 RNA. Four donors had plasma or serum available for paired testing. SARS-CoV-2 RNA was not detected from any tissue or plasma/serum sample tested. Based on these findings, risk of transmission through the transplantation of tissue types studied from SARS-CoV-2 infected donors is likely to be low.

## Introduction

The risk for transmission of SARS-CoV-2 through transplantation of allogeneic human tissues (tissues) is unknown. The identification of the angiotensin 2 converting enzyme (ACE-2) receptor as the primary receptor for virus to enter cells provides an imperfect guide to potential target tissues. There have been many reports describing the detection of SARS-CoV-2 RNA in tissues beyond the respiratory tract (Bradley 2020; Best 2021; Gaussen 2021; Penfield 2020; Trypsteen 2020). This includes reports that viral RNA (vRNA) is detectable in the blood of some patients, suggesting systemic viral dissemination (Yang 2020; Beyerstedt 2021) and broad organ involvement. While, historically, respiratory viruses were not thought to pose a significant risk for transmission via allograft tissue implantation, SARS-CoV-2 may behave differently due to this systemic dissemination. Considering the severity of illness, rapid community spread, and uncertainty surrounding tropism in human tissue, the American Association of Tissue Banks (AATB) issued guidance for screening and exclusion of donors who may be infected with SARS-CoV-2 (American Association of Tissue Banks 2020).

In the United States, human tissues for transplantation are regulated by the U.S. Food and Drug Administration (FDA) as human cells, tissues, and cellular and tissue-based products (HCT/Ps) under 21 CFR Part 1271. This study evaluated multiple different human tissues intended for transplantation, including bone, tendon, skin, fascia lata, vascular tissues, and heart valves. FDA requires all HCT/P donors to be screened and tested for relevant communicable disease agents or diseases for use in making a donor eligibility determination to exclude donors with the potential to transmit communicable diseases. Typically, when potential tissue donors are identified, recovery establishments perform a preliminary review for donor suitability, i.e., obtain donor information to identify any data that would obviously render the donor ineligible for donation according to standards established by FDA, AATB, and each tissue establishment. If a potential donor appears to be suitable for donation, tissues are then recovered, sent to the tissue processing facility where they are held or processed while completing the collection and review of donor medical and social history data.

Determining whether viral RNA (vRNA), and infectious virus, is regularly present in tissues typically used for transplantation collected from donors with a history of prior SARS-CoV-2 infection would better inform decisions regarding whether to exclude potential donors. AATB sponsored two studies to help evaluate the potential role of SARS-CoV-2 transmission in tissue transplantation. The first (Greenwald 2022) was to examine the risk of SARS-CoV-2 viremia in blood of deceased tissue donors, showing an incidence of RNAemia of approximately 1 in 1000. However, in that study, the results of SARS-CoV-2 RNA testing of donor nasopharyngeal (NP) swabs, if performed, was unknown, and tissues from these donors were not tested. To further characterize the risk of viral transmission and inform tissue safety policy, this retrospective study was performed to examine various tissues obtained from deceased donors whose NP swabs collected within 24 h of death tested positive for SARS-CoV-2 RNA for evidence of the SARS-CoV-2 via reverse transcriptase polymerase chain reaction testing (RT-PCR).

## Materials/methods

Between April 2020 and April 2021, tissue establishments identified stored, frozen human tissues collected from research-authorized deceased donors who had NP samples positive for SARS-CoV-2 RNA using nucleic acid amplification testing (NAT) for the presence of viral RNA (Table 1) and meeting all other study criteria (Table 2) for study inclusion. Research authorization was provided by the individual authorizing donation after death, or at the time of donation registration (first-person authorization), and all donor samples were anonymized by the establishment providing the research tissue and its associated recovery data. At the time of tissue collection, the donors were not suspected of being at risk of SARS-CoV-2 infection and there was no information available at the time tissues were recovered to indicate the donor would be ineligible for donation (Table 3). Tissue donor NP swabs were collected at the time of tissue recovery. Tissues were handled, stored, and processed according to the information provided in Table 4. None of the tissues underwent processing that included viral inactivation steps, and none of the antibiotics or antifungals used are known to have virucidal properties.

**Table 1:** Donor testing for SARS-CoV-2

Donor identification number	Sampling methodology	SARS-CoV-2 testing methodology	SARS-CoV-2 assay
D1	Nasopharyngeal Swab	RT-PCR^a^	BioGX SARS-CoV-2^b^
D2	Nasopharyngeal Swab	RT-PCR	BioGX SARS-CoV-2
D3	Nasopharyngeal Swab	RT-PCR	BioGX SARS-CoV-2
D4	Nasopharyngeal Swab	RT-PCR	Viracor SARS-CoV-2 Eurofins^c^
D5	Nasopharyngeal Swab	RT-PCR	Viracor SARS-CoV-2
D6	Nasopharyngeal Swab	RT-PCR	BioGX SARS-CoV-2
D7	Nasopharyngeal Swab	TMA^d^	Procleix SARS-CoV-2^e^
D8	Nasopharyngeal Swab	RT-PCR	BioGX SARS-CoV-2
D9	Nasopharyngeal Swab	RT-PCR	Viracor SARS-CoV-2
D10	Nasopharyngeal Swab	RT-PCR	BioGX SARS-CoV-2
D11	Nasopharyngeal Swab	RT-PCR	BioGX SARS-CoV-2
D12	Nasopharyngeal Swab	RT-PCR	BioGX SARS-CoV-2
D13	Nasopharyngeal Swab	TMA	Procleix SARS-CoV-2
D14	Nasopharyngeal Swab	TMA	Procleix SARS-CoV-2
D15	Nasopharyngeal Swab	TMA	Procleix SARS-CoV-2
D16	Nasopharyngeal Swab	TMA	Procleix SARS-CoV-2
D17	Nasopharyngeal Swab	TMA	Procleix SARS-CoV-2

^a^Reverse transcription-polymerase chain reaction

^b^Manufactured by Becton, Dickinson & Company

^c^Manufactured by Viracor Eurofins Clinical Diagnostics

^d^Transcription-mediated amplification

^e^Manufactured by Grifols

This table provides information about how the asymptomatic donors were determined to be SARS-CoV-2 positive. See Table 10 for more detail regarding assay performance characteristics.

**Table 2:** Study criteria

Inclusion criteria	Exclusion criteria
Donations collected within study timeframe, April 1, 2020–April 30, 2021	Donation occurred outside the study timeframe
At time of tissue collection, donor not known to have signs or symptoms of COVID-19 (nasopharyngeal swab test results would have been pending at the time of tissue collection), and no obvious reasons to consider the donor to be ineligible for donation according to FDA regulatory requirements	Donor known to have factors that would render them ineligible according to FDA regulatory requirements, or donor known or suspected to have SARS-CoV-2, otherwise the donor tissues would not have been collected
Donor nasopharyngeal swab sample found to be NAT-positive for SARS-CoV-2	
Donor authorized for research	Research authorization not obtained
Donations collected within the United States^a^	Donor demographic data not provided
Stored tissues available for testing	Stored tissues not available for testing
*Unknown*	
Whether the donor also was an organ donor	
Vaccination status of donors (the first COVID-19 vaccine was made available in the United States was on December 11, 2020)	

^a^Convenience sample based on willingness of tissue establishment to participate in the study

**Table 3:** Donor information

Manuscript donor identification number	Donor tissue recovery date	Age (years)	Gender	Race	Cause of death/mechanism of death	Circumstances/clinical course	Autopsy data	Histology data
D1	5/17/20	26	M	Caucasian	Drowning / Asphyxiation	Patient presented to ED via EMS in cardiac arrest after being dragged out of the water by a lifeguard at the beach. CPR was started on scene with unknown downtime	Near drowning leading to anoxic brain injury	Myocardium unremarkable
D2	5/27/20	50	M	Caucasian	Asystole cardiac arrest	Had been consuming alcohol all day on 5/26/20. Last seen alive 5/26 at 12:00. Wife returned home after 15:00 and found him unresponsive on floor. CPR initiated by wife. Asystole when EMS arrived. ACLS initiated by EMS and continued in the ER. Pronounced in the ER on 5/26/20 16:03	N/A^a^	N/A
D3	5/19/20	45	M	Caucasian	Hanging /asphyxiation	Patient found hanging from a tree by a bungee cord	Circumferential furrow around neck; ligature recovered; ETOH recovered from peripheral blood and vitreous fluid; isopropanol recovered from vitreous fluid	Heart pathology: hypereosinophilic myocytes and a foci of contraction band necrosis/coagulative myocytolysis; LAD 60% narrowed by atherosclerotic plaque
D4	4/21/20	69	F	Caucasian	Cardiac Arrest, acute respiratory failure with hypoxia, severe pulmonary hypertension	Admitted to hospital on 4/15/20 for shortness of breath. Diagnosed with pulmonary hypertension. She did not improve and died on 4/20/20 at 18:01. History of severe obesity	N/A	N/A
D5	4/23/20	46	M	Caucasian	Arrhythmia/asystole cardiac arrest	Found unresponsive in bed. EMS arrived. He has a shockable rhythm but then went into asystole. CPR and ACLS were initiated and continued in the ER. Pronounced in ER on 4/22/20 at 19:53	N/A	N/A
D6	6/4/20	47	M	Hispanic	Unknown	Last seen alive by wife 6/3/20 09:30. Pronounced dead at scene on 6/3/20 at 11:44	N/A	N/A
D7	8/19/20	53	M	Black	Heart Attack / Asystole cardiac arrest	Patient presented to ED via EMS for cardiac arrest. Per family patient complained of chest pain and was found unresponsive by bystanders with estimated downtime of 45 min. EMS arrived, started CPR, and patient remained in asystole	N/A	N/A
D8	5/17/20	40	F	Caucasian	Overdose	Patient had witnessed cardiac arrest (bardycardia– > PEA) and suspected oxycodone overdose (missing pill bottles) transferred to another hospital’s ICU for resp failure on ventilation, pontine hemorrhage and concern for cerebral edema	N/A	N/A
D9	4/17/20	57	M	Caucasian	Sudden Death	No fever or respiratory symptoms	N/A	Heart pathology normal
D10	5/11/20	25	M	Unknown	Sudden Death	Complained of headache/stomach pain, collapsed, pronounced in ER	N/A	Heart pathology normal
D11	5/21/20	33	M	Unknown	Sudden Death	Witnessed Arrest After Smoking Marijuana and Drank A Few Beers, EMS Called And Transported To ER And Pronounced	N/A	Heart pathology normal
D12	5/27/20	50	M	Caucasian	Sudden Death	Out of hospital cardiac arrest, history of asthma and unknown GI issues but otherwise healthy, no known complaints prior	N/A	Heart pathology normal
D13	9/21/20	5	F	Unknown	Trauma	Hit by vehicle	N/A	Heart pathology normal
D14	7/2/20	22	F	Unknown	Hanging / Asphyxiation	Attempted OD, then hung herself, no previous symptoms	N/A	Heart pathology normal
D15	11/4/20	47	F	Hispanic	Asystole cardiac arrest		N/A	N/A
D16	1/6/21	58	M	Caucasian	Asystole cardiac arrest		N/A	N/A
D17	4/29/21	51	M	Caucasian	Heart attack/asystole cardiac arrest	Circumstances surrounding death: Came in with STEMI, intubated, transferred from hospital for high-risk CABG. Coded multiple times. Family chose not to start ECMO and made comfort care; started the withdrawal process and died without further interventions	N/A	N/A

^a^Data not available because autopsy or histology were not performed

This table provides donor information including demographics, details surrounding the death, and any known autopsy or pathological data. Abbreviations: advanced cardiac life support (ACLS), cardiopulmonary resuscitation (CPR), coronary artery bypass graft (CABG), emergency department (ED), emergency room (ER), emergency medical services (EMS), pulseless electrical activity (PEA), ethyl alcohol (ETOH), extracorporeal membrane oxygenation (ECMO), female (F), gastrointestinal (GI), intensive care unit (ICU), left anterior descending (LAD), male (M), not applicable (N/A), overdose (OD), ST-elevation myocardial infarction (STEMI)

**Table 4:** Tissue handling and processing

Tissue type	Recovery technique	Transport to tissue bank	Processing at tissue bank	Transport to miller lab
Bone and tendon	Standard surgical techniques, *en bloc* for the tendons and disarticulated for the bone	Tissue placed in transport solution and transported on wet ice (1–10 °C) or dry ice (~ − 50 °C), arriving to the tissue establishment within 48–72 h after recovery	Stored frozen at − 20 °C or − 80 °C; no processing	Tissues shipped on dry ice (~ − 50 °C)
		Tissue is transported on wet ice (1–10 °C) for up to 36 h		
Cardiovascular (CV) tissues (i.e., artery, vein, heart valve)	Standard surgical techniques, en bloc for the vascular tissues, with no chemical agents applied to the tissue	Transported to the tissue bank packaged in normal saline, placed in a cooler on wet ice (1–10 °C) within 24 h of recovery	Dissected, disinfected with an antibiotic/antifungal cocktail, and cryopreserved in tissue culture media supplemented with 10% dimethylsulfoxide (cryoprotectant), 10% fetal calf serum and *cryopreserved* at − 1 °C per minute to at least − 50 °C and stored in the vapor phase of liquid nitrogen ~ − 150 °C	Tissues shipped on dry ice (~ − 50 °C)
			Dissected, disinfected with an antibiotic/ antifungal cocktail, stored − 80 °C freezer until shipped on dry ice	
Dermis	Standard surgical technique, surgical prep solution is used and then rinsed off during packaging stage	The tissue is then packaged in saline or RMPI, transported on wet ice (1–10 °C) for up to 36 h	Stored at 2–8 °C; no processing	Tissues shipped on dry ice (~ − 50 °C)
Blood Specimens	Collected after cessation of the heartbeat	After collection and separation, immediately stored at − 80 °C	Not applicable	Blood specimens shipped on dry ice (~ − 50 °C)

This table information provided about how tissues were handled and processed, including storage and transport temperatures. For some tissue types, there were differences among study participants in how tissues were handled, indicated by including different information within that category

Because there were finite lab resources available, amongst the available stored donor tissue identified, a convenience sample representing a variety of the tissue types available for transplantation was selected for testing. Human tissue and blood specimens were shipped frozen on dry ice to the Miller Laboratory at University of California Davis (ML/UCD) where tissues were kept in dry ice storage from receipt until they were prepared and tested between January 2021 and February 2022. All tissues and blood had been frozen once, and not thawed, at the time that they were shipped.

### Tissue preparation for sampling

The ML/UCD received 47 tissues from 17 donors, and 6 donor blood tubes (serum or plasma) from 4 of the 17 donors. Tissues were kept in dry ice storage until time of preparation for testing. Fascia lata, dermis, tendon, femur, and tibia were thawed overnight at 4 degrees Celsius. Cryopreserved cardiovascular tissues were thawed and rinsed per clinical instructions for use (cryopreservative solutions were properly removed), while the remaining cardiovascular tissues that were not control-rate frozen and without cryopreservation solution were thawed in the same manner as other tissues.

### Sample collection

Tissues were thawed to collect samples for testing. Three distinct sites/subsets (designated A, B, and C) were selected as samples for testing from all tissue sets (when possible). For bone, the subset sites included endosteum (A), periosteum (B) and cancellous bone (C), and for the other tissue types, the three subset sites were taken different areas of the selected tissue. For each distinct site, samples were collected in duplicate to allow ribonucleic acid (RNA) extraction using two different methodologies (i.e., Trizol or RNAeasy), and all RNA extracted from the samples was then suspended in RNAlater (Thermo Fisher Scientific/ Invitrogen) and snap-frozen on dry ice.

### RNA/cDNA processing

Tissue samples were thawed and immediately transferred from RNAlater preservation solution to a sterile bead beater tube containing 1 ml Trizol (Thermo Fisher Scientific/ Invitrogen) and a 7 mm stainless steel bead. Tissues were homogenized for 3–5 min. Homogenate was transferred to a new tube. RNA was extracted using either Trizol or the RNAeasy Mini Kit (Qiagen)*.* Frozen plasma and serum samples were thawed, and RNA was extracted using the Qiagen Ultrasens Virus Kit. One mg of complementary deoxyribonucleic acid (cDNA) was synthesized from the RNA extracted from each sample using Superscript IV (Thermo Fisher Scientific/ Invitrogen).

### Real-time PCR

Plasma and serum cDNA samples were tested immediately after cDNA synthesis using the Qiagen Ultrasens Virus Kit using primers and probes from Integrated DNA Technologies as described in Carroll (2022), Shaan Lakshmanappa (2021), and Deere (2021).

Tissue cDNA samples, which underwent two freeze–thaw cycles (one of which was part of the RNA assessment protocol), were run on the Quant Studio 6 Flex real-time PCR system (Qiagen) as described in Carroll (2022), Shaan Lakshmanappa (2021), and Deere (2021). All samples were tested in quadruplicate for both subgenomic RNA (sgRNA) and genomic nucleocapsid RNA (gRNA) of SARS-CoV-2, and in duplicate for the housekeeping gene Glyceraldehyde-3-phosphate dehydrogenase (GAPDH), which served as the positive control. Positive controls were included for all three targets on every plate. Human control RNA was used for GAPDH positive control. The human control RNA was also screened for sgRNA and gRNA (negative for both).

## Results

Results are reported across the 17 NP swab SARS-CoV-2-positive donors with donor identification designations as D1 through D17. Individual tissue sample identifiers are represented as the secondary number to the donor identification (i.e., D1.01, D1.02., etc.). Sample testing replicates for each tissue sample are identified with an alpha character (i.e., D1.01.A, D1.01.B, etc.).

Table 3 outlines the donor demographic and clinical data that were provided for the study. Twelve (12) male and five (5) female donors were evaluated; donors ranged in age from 5 to 69 years old with a median age of 47. Donor tissues were recovered between 4/17/2020 and 4/29/2021 with a median recovery date of 5/27/2020. Donations were recovered within the continental United States.

A range of circumstances and causes surrounding donor death were observed, including, but not limited to, sudden death, cardiac events, asphyxiation, and drug overdose. Cardiac histology data were limited; however, for the donors where histology data were provided, there were no indications of abnormal tissue pathology with the one exception being a donor showing contraction band necrosis/myocytolysis consistent with ischemic injury.

The results of all testing are provided in detail in Tables 5, 6, 7, 8, 9. No viral RNA was detected in any tissue or blood specimens provided.

**Table 5:** Blood testing results

Donor	Tissue classification	Tissue	Sample ID	Subset	Processing information	sgRNA	gRNA	Mean Ct-GAPDH
D11	Blood component	Plasma	D11.05.A	Single sample	No Processing	Negative	Negative	29.294
D15	Blood component	Plasma	D15.02.A	Single sample	No Processing	Negative	Negative	28.541
D15	Blood component	Serum	D15.03.A	Single sample	No Processing	Negative	Negative	27.248
D16	Blood component	Plasma	D16.02.A	Single sample	No Processing	Negative	Negative	32.780
D16	Blood component	Serum	D16.03.A	Single sample	No Processing	Negative	Negative	32.474
D17	Blood component	Plasma	D17.02.A	Single sample	No Processing	Negative	Negative	32.904

This table provides blood specimen (serum or plasma) results. All samples were tested in quadruplicate for both subgenomic RNA (sgRNA) and genomic nucleocapsid RNA (gRNA) of SARS-CoV-2, and in duplicate for the housekeeping gene Glyceraldehyde-3-phosphate dehydrogenase (GAPDH), which served as the positive control

**Table 6:** Cardiac tissue results

Donor	Tissue classification	Tissue	Sample ID	Subset	Processing information	sgRNA	gRNA	Mean Ct-GAPDH
D1	Cardiac	Ascending aorta	D1.07.A	Replicate sample	Dissection, Antibiotics, Cryopreserved	Negative	Negative	25.419
D1	Cardiac	Ascending aorta	D1.07.B	Replicate sample	Dissection, Antibiotics, Cryopreserved	Negative	Negative	24.132
D1	Cardiac	Ascending aorta	D1.07.C	Replicate sample	Dissection, Antibiotics, Cryopreserved	Negative	Negative	26.067
D1	Cardiac	Pulmonic valve	D1.08.A	Replicate sample	Dissection, Antibiotics, Cryopreserved	Negative	Negative	23.783
D1	Cardiac	Pulmonic valve	D1.08.B	Replicate Sample	Dissection, Antibiotics, Cryopreserved	Negative	Negative	23.797
D1	Cardiac	Pulmonic valve	D1.08.C	Replicate sample	Dissection, Antibiotics, Cryopreserved	Negative	Negative	24.65
D3	Cardiac	Pulmonic valve	D3.01.A	Replicate sample	Dissection, Antibiotics, Cryopreserved	Negative	Negative	19.782
D3	Cardiac	Pulmonic valve	D3.01.B	Replicate sample	Dissection, Antibiotics, Cryopreserved	Negative	Negative	19.532
D3	Cardiac	Pulmonic valve	D3.01.C	Replicate sample	Dissection, Antibiotics, Cryopreserved	Negative	Negative	18.766
D3	Cardiac	Pulmonary Artery (Segment)	D3.02.A	Replicate sample	Dissection, Antibiotics, Cryopreserved	Negative	Negative	24.639
D3	Cardiac	Pulmonary Artery (Segment)	D3.02.B	Replicate sample	Dissection, Antibiotics, Cryopreserved	Negative	Negative	24.663
D3	Cardiac	Pulmonary Artery (Segment)	D3.02.C	Replicate sample	Dissection, Antibiotics, Cryopreserved	Negative	Negative	22.758
D7	Cardiac	Pulmonary Artery (Segment)	D7.01.A	Replicate sample	Dissection, Antibiotics, Cryopreserved	Negative	Negative	23.99
D7	Cardiac	Pulmonary Artery (Segment)	D7.01.B	Replicate sample	Dissection, Antibiotics, Cryopreserved	Negative	Negative	27.149
D7	Cardiac	Pulmonary Artery (Segment)	D7.01.C	Replicate sample	Dissection, Antibiotics, Cryopreserved	Negative	Negative	26.764
D8	Cardiac	Hemi Pulmonary Artery (Right)	D8.01.A	Replicate sample	Dissection, Antibiotics, Cryopreserved	Negative	Negative	17.41
D8	Cardiac	Hemi Pulmonary Artery (Right)	D8.01.B	Replicate sample	Dissection, Antibiotics, Cryopreserved	Negative	Negative	21.218
D8	Cardiac	Hemi Pulmonary Artery (Right)	D8.01.C	Replicate sample	Dissection, Antibiotics, Cryopreserved	Negative	Negative	20.404
D9	Cardiac	Aortic valve	D9.01.A	Replicate sample	Dissection, Antibiotics, Flash Frozen	Negative	Negative	31.421
D9	Cardiac	Aortic valve	D9.01.B	Replicate sample	Dissection, Antibiotics, Flash Frozen	Negative	Negative	31.328
D9	Cardiac	Aortic valve	D9.01.C	Replicate sample	Dissection, Antibiotics, Flash Frozen	Negative	Negative	32.06
D9	Cardiac	Pulmonic valve	D9.02.A	Replicate sample	Dissection, Antibiotics, Flash Frozen	Negative	Negative	34.954
D9	Cardiac	Pulmonic valve	D9.02.B	Replicate sample	Dissection, Antibiotics, Flash Frozen	Negative	Negative	33.815
D9	Cardiac	Pulmonic valve	D9.02.C	Replicate sample	Dissection, Antibiotics, Flash Frozen	Negative	Negative	33.59
D10	Cardiac	Aortic valve	D10.03.A	Single sample	Dissection, Antibiotics, Flash Frozen	Negative	Negative	26.197
D10	Cardiac	Pulmonic valve	D10.04.A	Replicate sample	Dissection, Antibiotics, Flash Frozen	Negative	Negative	33.74
D10	Cardiac	Pulmonic valve	D10.04.B	Replicate sample	Dissection, Antibiotics, Flash Frozen	Negative	Negative	33.276
D10	Cardiac	Pulmonic valve	D10.04.C	Replicate sample	Dissection, Antibiotics, Flash Frozen	Negative	Negative	34.716
D11	Cardiac	Aortic valve	D11.04.A	Replicate sample	Dissection, Antibiotics, Flash Frozen	Negative	Negative	26.331
D11	Cardiac	Aortic valve	D11.04.B	Replicate sample	Dissection, Antibiotics, Flash Frozen	Negative	Negative	26.69
D11	Cardiac	Aortic valve	D11.04.C	Replicate sample	Dissection, Antibiotics, Flash Frozen	Negative	Negative	26.97
D12	Cardiac	Pulmonic valve	D12.01.A	Single sample	Dissection, Antibiotics, Flash Frozen	Negative	Negative	UND
D12	Cardiac	Aortic valve	D12.03.A	Replicate sample	Dissection, Antibiotics, Flash Frozen	Negative	Negative	23.434
D12	Cardiac	Aortic valve	D12.03.B	Replicate sample	Dissection, Antibiotics, Flash Frozen	Negative	Negative	23.519
D12	Cardiac	Aortic valve	D12.03.C	Replicate sample	Dissection, Antibiotics, Flash Frozen	Negative	Negative	32.084
D13	Cardiac	Aortic valve	D13.01.A	Replicate sample	Dissection, Antibiotics, Flash Frozen	Negative	Negative	25.848
D13	Cardiac	Aortic valve	D13.01.B	Replicate sample	Dissection, Antibiotics, Flash Frozen	Negative	Negative	26.596
D13	Cardiac	Aortic valve	D13.01.C	Replicate sample	Dissection, Antibiotics, Flash Frozen	Negative	Negative	26.523
D13	Cardiac	Pulmonic valve	D13.02.A	Replicate sample	Dissection, Antibiotics, Flash Frozen	Negative	Negative	25.703
D13	Cardiac	Pulmonic valve	D13.02.B	Replicate sample	Dissection, Antibiotics, Flash Frozen	Negative	Negative	26.293
D13	Cardiac	Pulmonic valve	D13.02.C	Replicate Sample	Dissection, Antibiotics, Flash Frozen	Negative	Negative	27.497
D14	Cardiac	Pulmonic valve	D14.01.A	Replicate Sample	Dissection, Antibiotics, Flash Frozen	Negative	Negative	32.882
D14	Cardiac	Pulmonic valve	D14.01.B	Replicate Sample	Dissection, Antibiotics, Flash Frozen	Negative	Negative	32.753
D14	Cardiac	Pulmonic valve	D14.01.C	Replicate sample	Dissection, Antibiotics, Flash Frozen	Negative	Negative	32.25
D14	Cardiac	Aortic valve	D14.02.A	Replicate sample	Dissection, Antibiotics, Flash Frozen	Negative	Negative	28.36
D14	Cardiac	Aortic valve	D14.02.B	Replicate sample	Dissection, Antibiotics, Flash Frozen	Negative	Negative	28.772
D14	Cardiac	Aortic valve	D14.02.C	Replicate sample	Dissection, Antibiotics, Flash Frozen	Negative	Negative	28.257

This table provides cardiac tissue results. All samples were tested in quadruplicate for both subgenomic RNA (sgRNA) and genomic nucleocapsid RNA (gRNA) of SARS-CoV-2, and in duplicate for the housekeeping gene Glyceraldehyde-3-phosphate dehydrogenase (GAPDH), which served as the positive control. UND indicates an undetected GAPDH result

**Table 7:** Vascular tissue results

Donor	Tissue classification	Tissue	Sample ID	Subset	Processing information	sgRNA	gRNA	Mean Ct-GAPDH
D1	Vascular	Femoral vein	D1.01.A	Replicate sample	Dissection, Antibiotics, Cryopreserved	Negative	Negative	22.949
D1	Vascular	Femoral vein	D1.01.B	Replicate sample	Dissection, Antibiotics, Cryopreserved	Negative	Negative	22.77
D1	Vascular	Femoral vein	D1.01.C	Replicate sample	Dissection, Antibiotics, Cryopreserved	Negative	Negative	24.587
D1	Vascular	Femoral artery	D1.02.A	Replicate sample	Dissection, Antibiotics, Cryopreserved	Negative	Negative	24.451
D1	Vascular	Femoral artery	D1.02.B	Replicate sample	Dissection, Antibiotics, Cryopreserved	Negative	Negative	23.93
D1	Vascular	Femoral artery	D1.02.C	Replicate sample	Dissection, Antibiotics, Cryopreserved	Negative	Negative	23.788
D1	Vascular	Femoral vein	D1.03.A	Replicate sample	Dissection, Antibiotics, Cryopreserved	Negative	Negative	17.776
D1	Vascular	Femoral vein	D1.03.B	Replicate sample	Dissection, Antibiotics, Cryopreserved	Negative	Negative	17.863
D1	Vascular	Femoral vein	D1.03.C	Replicate sample	Dissection, Antibiotics, Cryopreserved	Negative	Negative	17.4
D1	Vascular	Femoral artery	D1.04.A	Replicate sample	Dissection, Antibiotics, Cryopreserved	Negative	Negative	18.99
D1	Vascular	Femoral artery	D1.04.B	Replicate sample	Dissection, Antibiotics, Cryopreserved	Negative	Negative	19.868
D1	Vascular	Femoral artery	D1.04.C	Replicate sample	Dissection, Antibiotics, Cryopreserved	Negative	Negative	18.969
D1	Vascular	Saphenous vein	D1.05.A	Replicate sample	Dissection, Antibiotics, Cryopreserved	Negative	Negative	17.306
D1	Vascular	Saphenous vein	D1.05.B	Replicate sample	Dissection, Antibiotics, Cryopreserved	Negative	Negative	17.677
D1	Vascular	Saphenous vein	D1.05.C	Replicate sample	Dissection, Antibiotics, Cryopreserved	Negative	Negative	18.172
D1	Vascular	Aortoiliac artery	D1.06.A	Replicate sample	Dissection, Antibiotics, Cryopreserved	Negative	Negative	21.214
D1	Vascular	Aortoiliac artery	D1.06.B	Replicate sample	Dissection, Antibiotics, Cryopreserved	Negative	Negative	18.576
D1	Vascular	Aortoiliac artery	D1.06.C	Replicate sample	Dissection, Antibiotics, Cryopreserved	Negative	Negative	20.055
D3	Vascular	Saphenous vein	D3.03.A	Replicate sample	Dissection, Antibiotics, Cryopreserved	Negative	Negative	23.529
D3	Vascular	Saphenous vein	D3.03.B	Replicate sample	Dissection, Antibiotics, Cryopreserved	Negative	Negative	23.215
D3	Vascular	Saphenous vein	D3.03.C	Replicate sample	Dissection, Antibiotics, Cryopreserved	Negative	Negative	21.323
D8	Vascular	Femoral vein	D8.01.A	Replicate sample	Dissection, Antibiotics, Cryopreserved	Negative	Negative	18.379
D8	Vascular	Femoral vein	D8.01.B	Replicate sample	Dissection, Antibiotics, Cryopreserved	Negative	Negative	20.828
D8	Vascular	Femoral vein	D8.01.C	Replicate sample	Dissection, Antibiotics, Cryopreserved	Negative	Negative	25.254
D8	Vascular	Femoral vein	D8.03.A	Replicate sample	Dissection, Antibiotics, Cryopreserved	Negative	Negative	18.597
D8	Vascular	Femoral vein	D8.03.B	Replicate sample	Dissection, Antibiotics, Cryopreserved	Negative	Negative	19.127
D8	Vascular	Femoral vein	D8.03.C	Replicate sample	Dissection, Antibiotics, Cryopreserved	Negative	Negative	18.467
D10	Vascular	Saphenous vein	D10.01.A	Replicate sample	Dissection, Antibiotics, Flash Frozen	Negative	Negative	22.387
D10	Vascular	Saphenous vein	D10.01.B	Replicate sample	Dissection, Antibiotics, Flash Frozen	Negative	Negative	22.695
D10	Vascular	Saphenous vein	D10.01.C	Replicate sample	Dissection, Antibiotics, Flash Frozen	Negative	Negative	21.536
D10	Vascular	Femoral artery	D10.02.A	Replicate sample	Dissection, Antibiotics, Flash Frozen	Negative	Negative	UND
D10	Vascular	Femoral artery	D10.02.B	Replicate sample	Dissection, Antibiotics, Flash Frozen	Negative	Negative	31.228
D10	Vascular	Femoral artery	D10.02.C	Replicate sample	Dissection, Antibiotics, Flash Frozen	Negative	Negative	27.105
D11	Vascular	Aortoiliac artery	D11.01.A	Replicate sample	Dissection, Antibiotics, Flash Frozen	Negative	Negative	24.188
D11	Vascular	Aortoiliac Artery	D11.01.B	Replicate sample	Dissection, Antibiotics, Flash Frozen	Negative	Negative	24.988
D11	Vascular	Aortoiliac Artery	D11.01.C	Replicate sample	Dissection, Antibiotics, Flash Frozen	Negative	Negative	25.912
D11	Vascular	Saphenous vein	D11.02.A	Replicate sample	Dissection, Antibiotics, Flash Frozen	Negative	Negative	24.626
D11	Vascular	Saphenous vein	D11.02.B	Replicate sample	Dissection, Antibiotics, Flash Frozen	Negative	Negative	23.023
D11	Vascular	Saphenous vein	D11.02.C	Replicate sample	Dissection, Antibiotics, Flash Frozen	Negative	Negative	22.853
D11	Vascular	Femoral artery	D11.03.A	Replicate sample	Dissection, Antibiotics, Flash Frozen	Negative	Negative	26.37
D11	Vascular	Femoral artery	D11.03.B	Replicate sample	Dissection, Antibiotics, Flash Frozen	Negative	Negative	27.438
D11	Vascular	Femoral artery	D11.03.C	Replicate sample	Dissection, Antibiotics, Flash Frozen	Negative	Negative	26.736
D12	Vascular	Saphenous vein	D12.02.A	Replicate sample	Dissection, Antibiotics, Flash Frozen	Negative	Negative	27.649
D12	Vascular	Saphenous vein	D12.02.B	Replicate sample	Dissection, Antibiotics, Flash Frozen	Negative	Negative	UND
D12	Vascular	Saphenous vein	D12.02.C	Replicate sample	Dissection, Antibiotics, Flash Frozen	Negative	Negative	26.856

This table provides vascular tissue results. All samples were tested in quadruplicate for both subgenomic RNA (sgRNA) and genomic nucleocapsid RNA (gRNA) of SARS-CoV-2, and in duplicate for the housekeeping gene Glyceraldehyde-3-phosphate dehydrogenase (GAPDH), which served as the positive control. UND indicates an undetected GAPDH result

**Table 8:** Musculoskeletal tissue results

Donor	Tissue classification	Tissue	Sample ID	Subset	Processing information	sgN	wtN	Mean Ct-GAPDH
D2	Musculoskeletal	Femur (Left)	D2.01.A	Endosteum	No processing	Negative	Negative	28.061
D2	Musculoskeletal	Femur (Left)	D2.01.B	Periosteum	No processing	Negative	Negative	28.223
D2	Musculoskeletal	Femur (Left)	D2.01.C	Cancellous Bone	No processing	Negative	Negative	24.171
D2	Musculoskeletal	Gracilis Tendon (Left)	D2.02.A	Replicate sample	No processing	Negative	Negative	29.544
D2	Musculoskeletal	Gracilis Tendon (Left)	D2.02.B	Replicate sample	No processing	Negative	Negative	29.19
D2	Musculoskeletal	Gracilis Tendon (Left)	D2.02.C	Replicate sample	No processing	Negative	Negative	29.51
D2	Musculoskeletal	Tibia (Left)	D2.05.A	Endosteum	No Processing	Negative	Negative	28.998
D2	Musculoskeletal	Tibia (Left)	D2.05.B	Periosteum	No processing	Negative	Negative	24.958
D2	Musculoskeletal	Tibia (Left)	D2.05.C	Cancellous bone	No processing	Negative	Negative	29.583
D4	Musculoskeletal	Semitendinosus Tendon (Right)	D4.01.A	Replicate sample	No processing	Negative	Negative	29.263
D4	Musculoskeletal	Semitendinosus Tendon (Right)	D4.01.B	Replicate sample	No processing	Negative	Negative	28.705
D4	Musculoskeletal	Semitendinosus Tendon (Right)	D4.01.C	Replicate sample	No processing	Negative	Negative	30.03
D4	Musculoskeletal	Tibia (Left)	D4.02.A	Endosteum	No processing	Negative	Negative	30.557
D4	Musculoskeletal	Tibia (Left)	D4.02.B	Periosteum	No processing	Negative	Negative	29.521
D4	Musculoskeletal	Tibia (Left)	D4.02.C	Cancellous bone	No processing	Negative	Negative	23.819
D4	Musculoskeletal	Femur (Left)	D4.03.A	Endosteum	No processing	Negative	Negative	26.66
D4	Musculoskeletal	Femur (Left)	D4.03.B	Periosteum	No processing	Negative	Negative	30.227
D4	Musculoskeletal	Femur (Left)	D4.03.C	Cancellous bone	No processing	Negative	Negative	23.393
D5	Musculoskeletal	Semitendinosus Tendon (Right)	D5.01.A	Replicate sample	No processing	Negative	Negative	30.722
D5	Musculoskeletal	Semitendinosus Tendon (Right)	D5.01.B	Replicate sample	No processing	Negative	Negative	28.714
D5	Musculoskeletal	Semitendinosus Tendon (Right)	D5.01.C	Replicate sample	No processing	Negative	Negative	29.516
D5	Musculoskeletal	Tibia (Right)	D5.02.A	Endosteum	No processing	Negative	Negative	22.375
D5	Musculoskeletal	Tibia (Right)	D5.02.B	Periosteum	No processing	Negative	Negative	21.736
D5	Musculoskeletal	Tibia (Right)	D5.02.C	Cancellous bone	No processing	Negative	Negative	22.038
D6	Musculoskeletal	Achilles Tendon (Right)	D6.01.A	Replicate sample	No processing	Negative	Negative	29.536
D6	Musculoskeletal	Achilles Tendon (Right)	D6.01.B	Replicate sample	No processing	Negative	Negative	29.911
D6	Musculoskeletal	Achilles Tendon (Right)	D6.01.C	Replicate sample	No processing	Negative	Negative	24.643
D6	Musculoskeletal	Humerus (Right)	D6.02.A	Endosteum	No processing	Negative	Negative	24.452
D6	Musculoskeletal	Humerus (Right)	D6.02.B	Periosteum	No processing	Negative	Negative	27.349
D6	Musculoskeletal	Humerus (Right)	D6.02.C	Cancellous bone	No processing	Negative	Negative	21.908
D15	Musculoskeletal	Fascia Lata	D15.01.A	Replicate sample	Dissection, saline rinse	Negative	Negative	27.013
D15	Musculoskeletal	Fascia Lata	D15.01.B	Replicate sample	Dissection, Saline Rinse	Negative	Negative	26.887
D15	Musculoskeletal	Fascia Lata	D15.01.C	Replicate sample	Dissection, saline rinse	Negative	Negative	29.475
D16	Musculoskeletal	Fascia Lata	D16.01.A	Replicate sample	Dissection, saline rinse	Negative	Negative	27.635
D16	Musculoskeletal	Fascia Lata	D16.01.B	Replicate sample	Dissection, Saline Rinse	Negative	Negative	29.245
D16	Musculoskeletal	Fascia Lata	D16.01.C	Replicate sample	dissection, saline rinse	Negative	Negative	28.883
D17	Musculoskeletal	Fascia Lata	D17.01.A	Replicate sample	Dissection, saline rinse	Negative	Negative	29.467
D17	Musculoskeletal	Fascia Lata	D17.01.B	Replicate sample	Dissection, saline rinse	Negative	Negative	27.725
D17	Musculoskeletal	Fascia Lata	D17.01.C	Replicate sample	Dissection, saline rinse	Negative	Negative	28.083

This table provides musculoskeletal tissue results. All samples were tested in quadruplicate for both subgenomic RNA (sgRNA) and genomic nucleocapsid RNA (gRNA) of SARS-CoV-2, and in duplicate for the housekeeping gene Glyceraldehyde-3-phosphate dehydrogenase (GAPDH), which served as the positive control

**Table 9:** Dermis tissue results

Donor	Tissue classification	Tissue	Sample ID	Subset	Processing information	sgRNA	gRNA	Mean Ct-GAPDH
D2	Dermis	Dermis (Posterior)	D2.03.A	Replicate sample	No processing	Negative	Negative	24.045
D2	Dermis	Dermis (Posterior)	D2.03.B	Replicate sample	No processing	Negative	Negative	24.972
D2	Dermis	Dermis (Posterior)	D2.03.C	Replicate sample	No processing	Negative	Negative	24.736
D2	Dermis	Dermis (Anterior)	D2.04.A	Replicate sample	No Processing	Negative	Negative	24.859
D2	Dermis	Dermis (Anterior)	D2.04.A	Replicate sample	No processing	Negative	Negative	24.133
D2	Dermis	Dermis (Anterior)	D2.04.C	Replicate sample	No processing	Negative	Negative	24.514

This table provides dermis results. All samples were tested in quadruplicate for both subgenomic RNA (sgRNA) and genomic nucleocapsid RNA (gRNA) of SARS-CoV-2, and in duplicate for the housekeeping gene Glyceraldehyde-3-phosphate dehydrogenase (GAPDH), which served as the positive control

Testing results for cadaveric (post cessation of heartbeat) blood available for D11 (plasma only) and D15–D17 (plasma and serum) are outlined in Table 5 and indicate negative real-time PCR cycle threshold (Ct) results for nucleocapsid sgRNA and gRNA. The remaining donors did not have cadaveric blood samples available for testing in this study.

Cardiac and vascular tissue type RNA target results are included in Tables 6 and 7. Cardiac tissues, including pulmonic and aortic valves, ascending aorta, and pulmonary arteries were tested from ten (10) donors. Vascular tissues, femoral veins and arteries, saphenous veins, and aortoiliac arteries, were tested from six (6) donors. Cardiac tissue was negative for sgRNA and gRNA using real-time PCR, while the mean GAPDH Ct values ranged from 17.41 to 34.954. Vascular tissue sgRNA and gRNA targets were negative using real-time PCR with mean Ct-GAPDH values ranging from 17.4 to 27.736.

Table 8 outlines musculoskeletal (MS) tissue RNA target results. Seven (7) donors tested both bone and soft tissues, femur, tibia, humerus, gracilis tendon, semitendinosus tendon, and fascia lata. Viral RNA targets (sgRNA and gRNA) were negative for all MS tissue types. Mean Ct-GAPDH values ranged from 21.908 to 30.722.

Table 9 outlines dermis tissue RNA target results from one (1) donor where this tissue type was procured. Dermis tissue sgRNA and gRNA targets were negative using real-time PCR. Mean Ct-GADPH values ranged between 24.045 and 24.972.

## Discussion

This study tested human tissues, intended for transplantation, for evidence of SARS-CoV-2. There was a focus on oversampling for tissue types that tend to be minimally processed and tropic for the virus, as those have been the tissue types most likely to transmit infection in past outbreaks involving a variety of pathogens (Tugwell 2005; CDC 2011; Schwartz 2022; Lu 2018; Greenwald 2012). We did not detect SARS-CoV-2 sgRNA or gRNA in any of the samples tested. However, we did detect GAPDH mRNA in all samples, except for one replicate of each of two samples of cardiovascular cissue, indicating that the RNA extraction and PCR methods used were valid. Further, the level of GAPDH in a sample was consistent with the cellularity of that sample. Thus, the samples that contained very few cells, tendon, heart valves, had lower levels of GAPDH (higher CT values) than more cellular samples from the dermis and vasculature. We quantified sgRNA because SARS-CoV-2 sgRNA is a good surrogate marker of infectivity (Santos Bravo 2022).

Many early exploratory studies detected SARS-CoV-2 by various testing methods in multiple organs and biospecimens, only few of which were designed to determine whether live virus was present (Bradley 2020; Best 2021; Gaussen 2021; Penfield 2020; Trypsteen 2020). While the ACE-2 receptor is required for cellular infection, the extent to which cells with ACE-2 receptors become infected with SARS-CoV-2 remains unclear. Furthermore, it is not clear whether end-organ damage observed is a result of direct viral activity or if it was immune-mediated, while emerging evidence indicates much of the observed damage outside of the respiratory system is likely immune-mediated (Merad 2022).

In October 2020, Trypsteen and colleagues reviewed available data regarding SARS-CoV-2 tropism. At that time, only respiratory and GI tract samples (mostly stool) demonstrated evidence that viral particles from a biopsy were capable of reinfecting target cells *in-vitro*. For cardiac tissue, there was discussion of the high level of acute cardiac injury that occurred in individuals hospitalized in the ICU with COVID-19, and the need to further investigate whether this was due to direct viral effects versus immune-mediated damage. Furthermore, some studies detected viral particles in or around cardiac tissue, but not within the myocytes. They concluded that the evidence at that time suggested immune mediated injury is more likely the culprit for the observed cardiac damage, but additional studies would be required.

In July 2021, Gaussen and colleagues reviewed literature for evidence of SARS-CoV-2 transmissibility via cell, tissue, and organ transplantation. Most studies included in this review article looked for evidence of the virus (e.g., NAT testing, direct visualization) while only few performed viral infectivity assays. As presented in the Gaussen review article, the evidence was strong for SARS-CoV-2 presence in lungs and the respiratory tract, both by NAT testing and some viral infectivity assays. Kidneys had some intermittent positive NAT results amongst tissues obtained from individuals who died of COVID-19, while only one study had found evidence of a small subset of kidneys having evidence of viral infectivity (Braun 2020). Ocular tissue testing largely did not have positive NAT in SARS-CoV-2-infected individuals, but one cited study showed NAT positivity and positive viral infectivity in an individual with COVID and bilateral conjunctivitis at the time of death (Colavita 2020). Studies including heart tissue demonstrated mostly histopathological evidence of damage with only rare instances of myocarditis. There was one cited study where testing of heart tissue from 39 individuals deceased from COVID demonstrated PCR positive results in 24/39 individuals and among those, 5 individuals with the highest viral load also demonstrated viral replication determined by cDNA synthesis (Lindner 2020).

Our study did not include donations of birth tissue (e.g., placenta, amniotic membrane) or reproductive tissue (i.e., semen or oocytes). Best and colleagues evaluated the semen of men who were diagnosed with acute SARS-CoV- 2 by RT-PCR testing of NP swab specimens. Among the 30 semen samples provided by subjects during the 11–64 days after testing positive for SARS-CoV-2 infection, 16 semen samples were tested for SARS-CoV-2 by RT-PCR (in addition to the semen analysis performed on all samples) and found to be negative. Penfeld et al. (2020) reported a study of 32 pregnant patients who delivered at the time of being diagnosed with COVID-19 infection. They obtained placental swabs of 11 of those patients, and found 3 of those 11 placental swabs to be positive for SARS-CoV-2 by RT-PCR—the positive placental specimens were found in women with severe COVID disease at the time of delivery. Follow-up studies to verify whether positive results on placenta represent viable virus should be performed.

To date, there has been no known transmission via tissue or ocular transplantation (FDA 2021) or blood transfusion (FDA 2022), and the only verified organ transmission was via lung transplantation (Kaul et al. 2021). In a study testing blood specimens from deceased tissue donors without COVID-19 symptoms, the rate of SARS-CoV-2 NAT-positive results (about 1 in 1000) was found, while infectivity data are unavailable (Greenwald 2022). Considering that, through Spring 2022, most tissue establishments have excluded donors with positive NP swab results for SARS-CoV-2, the true risk of transmission is difficult to measure. Although no transmission events have been reported through human tissue, since there are recognized challenges of identifying donor-derived transmission amongst allograft recipients (Greenwald 2012), the actual risk of transmission via tissues cannot be reliably confirmed by absence of transmission alone.

In our study, amongst the 17 donors who died of causes not known to be related to COVID but were found to have positive NP swabs, 45 tissue samples and 6 blood samples were tested by two different methodologies and no SARS-CoV-2 RNA was detected. Follow-up viral culture to determine infectivity was therefore not possible. It is notable that in the few studies where replication-competent SARS-CoV-2 was detected in non-respiratory organs and tissues, the virus was found only intermittently and in tissues or organs from individuals who were known to have died of COVID-19 infection (Gaussen 2021). Available evidence therefore indicates that asymptomatic tissue donors, regardless of their SARS-CoV-2 NP swab test results, are unlikely to have SARS-CoV-2 present in their tissues, much less a replication-competent virus. While it is notable that among the 17 donors in our study, 10 died suddenly of unknown causes (often attributed to “cardiac death” or “sudden cardiac death”), and another donor died of ST-elevation myocardial infarction (STEMI), there is not enough information available to draw any conclusions about the potential role of undiagnosed COVID-19, including hypercoagulability, in the donor deaths.

### Our study has limitations

First, our study was not a random sample of donors who tested positive by NP swabs for SARS-CoV-2, but rather a convenience sample of donors provided by participating US tissue processors in which tissues were recovered, available, and met acceptability criteria for recovery. As such, included donors were most likely not severely ill with COVID-19, and perhaps therefore less likely to have disseminated infection with the virus to involve recovered tissues. No viral RNA was detected in blood from donors tested in this study, but this subsample and the overall study sample size were small. Second, the donor nasopharyngeal swab testing was performed using assays under Emergency Use Authorization since there were no available FDA-cleared or approved test kits, see Table 10 for information on assay performance characteristics. Third, tissue storage (Auer et al. 2014; Bao 2013), freeze thaw cycles (Dzung 2021; Botling 2009), and any minimal processing performed (e.g., rinsing) could have impacted results (Schilling-Loeffler 2022; Nogueira 2022). The bone and tendon were not rinsed prior to testing and thus represent a worst case for viral load. The dermis, heart, and vascular tissue were rinsed either in transport solution (all) or during processing (heart and veins). As the virus is an intracellular pathogen, it will not be passively rinsed off the tissue; however, cells such as endothelium that may contain virus could be dislodged. These rinses are representative of processing for human tissues and thus representative of the potential viral load after rinsing. Finally, the effect of different SARS-CoV-2 variants circulating during the study, with possible differences in virulence and tropism, are unknown since genotyping was not available.

**Table 10:** Performance characteristics of EUA assays used in testing donor NP specimens

Assay (manufacturer)	Sample type	Sensitivity	Specificity	Limit of detection (LOD)^1^
BioGX^2^ SARS-CoV-2 (Becton, Dickinson & Company)	NP Swab	19/19^a^ positive at ~ 1–2 × LOD; 10/10 at ~ 3–5 × LOD	30/30 negative	40 genome equivalents (GE)/mL
Viracor^3^ SARS-CoV-2 (Viracor Eurofins Clinical Diagnostics)	NP Swab	20/20 positive at ~ 2 × LOD	30/30 negative	73 copies/mL
Procleix^4^ SARS-CoV-2 (Grifols)	NP Swab	Positive Percent Agreement: 30/30; 100% (95% CI: 88.65–100.00%)^*^	Negative Percent Agreement: 30/30; 100% (95% CI: 88.65–100.00%)^*^	60 copies/mL

^a^During screening one retrospective nasopharyngeal swab clinical sample resulted in an UND for N1 and as a result was removed from data analysis

^*^Two-sided 95% score confidence intervals

^1^Lowest concentration of genomic RNA from SARS-CoV-2 that can be reproducibly distinguished from negative samples ≥ 95% of the time

^2^EUA data available via FDA at https://www.fda.gov/medical-devices/emergency-use-authorizations-medical-devices/historical-information-about-device-emergency-use-authorizations and https://www.fda.gov/media/136653/download

^3^EUA data available via FDA at https://www.fda.gov/media/143069/download?attachment

^4^EUA data available via FDA at https://www.fda.gov/media/145938/download and additional information provided in Sauleda et al. (2022) and Bakkour (2021)

Table 10 provides performance information available at the time of testing for EUA assays used to test donor nasopharyngeal swabs for SARS-CoV-2.

In conclusion, this study did not detect SARS-CoV-2 in MS, vascular, and skin types of human tissue collected from donors with positive NP swabs. There were no data collected on birth or reproductive tissue. The preponderance of evidence, from this research data and others, is reassuring that the likelihood of transmission of SARS-CoV-2 through the human tissue studied is likely low.

## Data availability statement

All data generated or analyzed during this study are included in this published article.
